# Women and the Conjoint Diploma

**Published:** 1907-04-06

**Authors:** 


### Women and the Conjoint Diploma.

At the last ordinary meeting of the Council of the Royal College of Surgeons of England a petition was presented in favour of the admission of women to the examinations conducted by the College for its surgical diplomas. The petition had the official support of the London School of Medicine for Women, and it carried a list of nearly 3,000 signatures. None of the signatures were those of women practitioners or of lay persons other than members officially connected with the Women's School. Hence it is a simple statement of fact to say that the petition represents the opinion of a considerable section of the male members of the profession. As such, it will doubtless receive the careful consideration of the College authorities. Those who support its prayer will be the first to recognise that the occasion is not one for anything in the shape of ill-considered haste. They believe that the conclusion which the petition embodies rests upon arguments sound alike in justice and policy, and they are more than content to await the issue of a candid and thoughtful judgment. At the same time it must be recognised that the petitioners are seriously in earnest. They believe themselves to be dealing with a distinct and substantial injustice, and they are determined to continue their efforts to remedy this.

The position occupied by the Royal Colleges of Physicians and Surgeons in England in refusing admission to women students would seem, on the face of it, to need justification. It is true that a similar attitude is adopted by some of the English universities, but with these exceptions almost all the universities and medical examining boards of the United Kingdom admit women to their examinations on the same terms as men. And it would be difficult to find any responsible person who would say that any harmful effect has followed this regulation. Hence the English Royal Colleges are not being asked to take a step in an entirely new and unexplored direction. Precedents and experiences may be found in each of the three Kingdoms, and the onus of proof, therefore, rests not on those who support, but on those who oppose the suggested movement. When, omitting for the time being the question of the universities, the conjoint colleges both in Scotland and Ireland and the London Society of Apothecaries all open their portals to women candidates, there is a *primâ facie* case in favour of the same policy on the part of the English Conjoint Colleges. It must here also be remembered that most of the examining authorities who now admit women have come to do so as the result of a deliberate and carefully considered decision. At no very remote date no woman in this country could obtain a diploma or license to practise medicine. When the change was first proposed it called forth all those gloomy prophecies which invariably make their appearance on the suggestion of some new departure, and which have so often been falsified by the course of events. The admission of women to the profession, it may be allowed, was a consi[illegible]erable change, and one concerning which diffe[illegible]t opinions might reasonably be held. If doubts [illegible] this point still exist, no one proposes to act u[illegible] them. But the question with which we are now concerned is one of much smaller proportions. It is not whether women shall or shall not be allowed to enter the profession of medicine, but merely whether a particular avenue to the profession shall or shall not remain closed to them. Whatever the decision, it will not alter by a single unit the number of women students or women practitioners. For London students more particularly the avenue is a convenient one, as is shown by the number of men who select it. Why should the women students in England be deprived of the opportunity enjoyed by the men students, and possessed also in substance by members of their own sex in other parts of the Kingdom? In this simple question lies the issue of the present debate, and it rests with the opposition—a difficult task, we imagine—to produce reasons in support of the peculiar and exceptional position which they wish the English Colleges to occupy.

The Council of the Royal College of Surgeons who have now to deal with the petition thus formally presented to them, though directly charged with the interests of the College, will, we doubt not, regard the question from a broad standpoint. They will certainly have to meet an opposition which will reflect both ancient prejudices and the fear of possible further demands. Of course, they must pay due deference to the views of their constituents. But it is beyond question that there is a growing opinion in favour of the admission of women and an increasing sense of the impossibility of justifying an attitude of the English colleges which is in flat contradiction to the practice of the majority of the universities and of all the other licensing authorities. For the credit of the College, not less than in the interests of our women students in London, we trust the prayer of the petition will be granted.

